# Supplementary material for: The genomic landscape of 85 advanced neuroendocrine neoplasms reveals subtype-heterogeneity and potential therapeutic targets
Source: Nat Commun. 2021 Jul 29;12:4612. doi: 10.1038/s41467-021-24812-3 (PMC8322054; doi:10.1038/s41467-021-24812-3)
Supplement: Supplementary file 1 — Supplementary Information [file 41467_2021_24812_MOESM1_ESM.pdf]

Supplementary Information

The genomic landscape of 85 advanced neuroendocrine neoplasms reveals subtype-heterogeneity and potential therapeutic targets

van Riet et al.

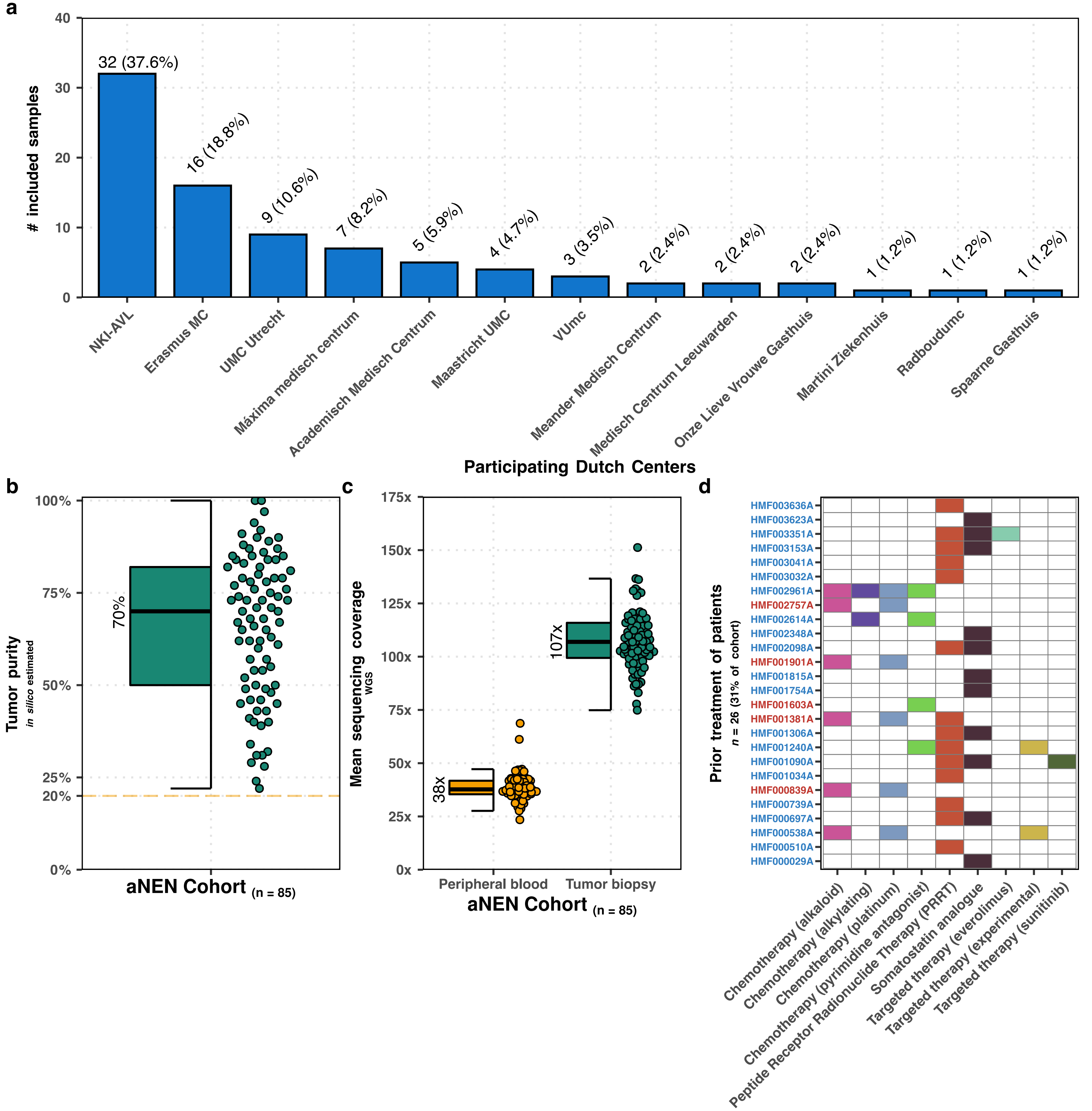

Suppl. Figure 1 - Overview of sequencing characteristics and selected biopsies.
a) Absolute and relative frequencies of distinct included patients (n = 85) in the CPCT-02 aNEN cohort per participating center within the Netherlands.
b) Boxplot with individual data points of the estimated (in silico) tumor cell percentages based on the whole genome sequencing data (n = 85) with observed median displayed. The cut-off of 20% tumor cell percentage is shown by an orange line. The median, interquartile range (IQR), and 1.5x the IQR are represented by a solid black line, box, and whiskers, respectively.
c) Boxplot with individual data points of the mean read-coverages (WGS) of the peripheral blood (reference; orange) and biopsy tissues (green) with observed median per variable displayed. The median, interquartile range (IQR), and 1.5x the IQR are represented by a solid black line, box, and whiskers, respectively.
d) Type of prior treatment per aNEN, if applicable (n = 26 out of 85 patients). Patients with aNET are highlighted in blue on the y-axis whilst patients with aNEN are highlighted in red on the y-axis.

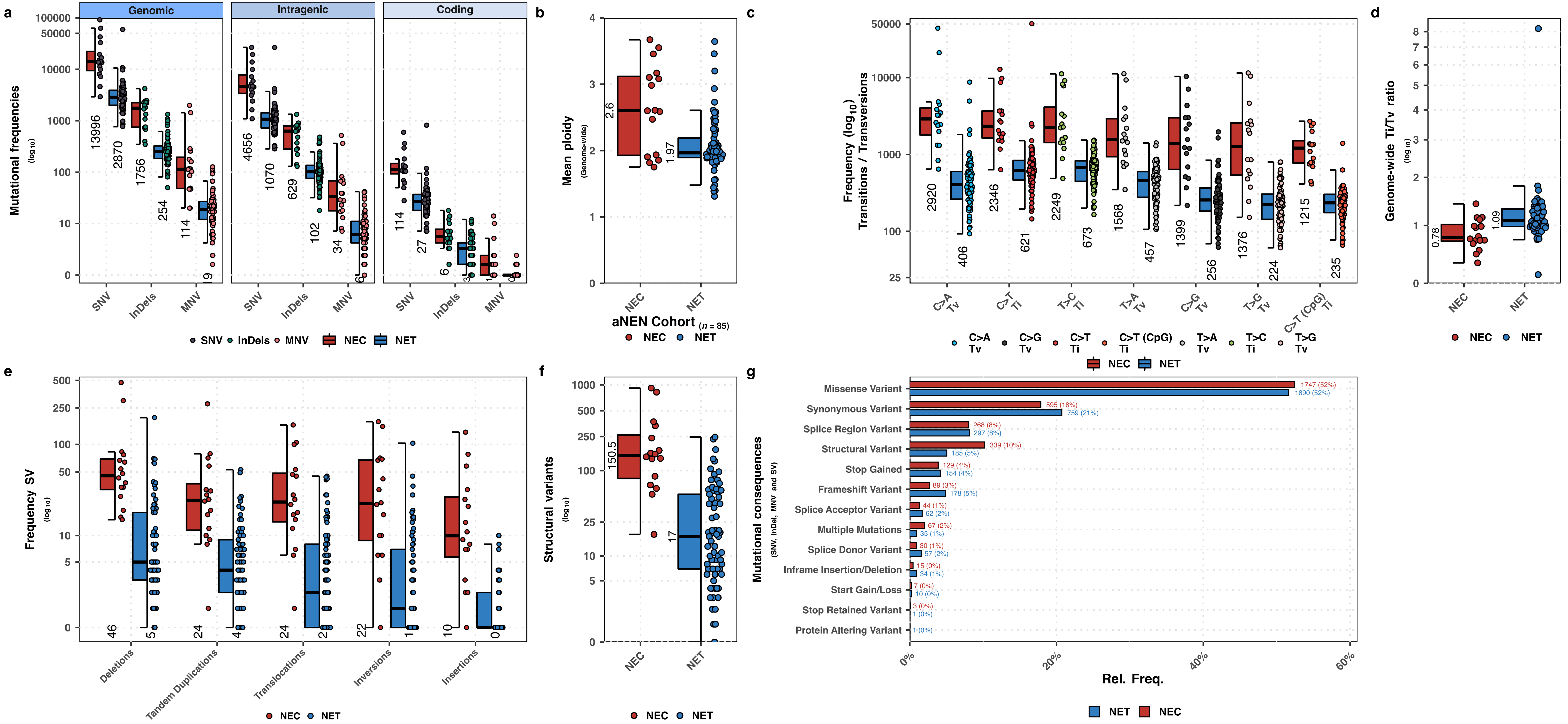

Suppl. Figure 2 - Overview of mutational landscape, categorized per differentiation grade.

a) Number of SNV (gray), InDels (green), MNV (salmon) per whole-genome sequenced sample (n = 85) over three resolutions; genome-wide, within intragenic regions and within coding regions with observed median per variable displayed. Data is categorized on aNEC / aNET status. The median, interquartile range (IQR), and 1.5x the IQR are represented by a solid black line, box, and whiskers, respectively.

b) Boxplots of mean genome-wide tumor ploidy based on all autosomal chromosomes with observed median displayed. Data is categorized on aNEC / aNET status.

c) Type of genome-wide SNVs. Transition (Ti) and transversion (Tv), with a special attention for C to T (Ti) in CpG context, are indicated per sample with observed median per variable displayed. Data is categorized on aNEC / aNET status. The median, interquartile range (IQR), and 1.5x the IQR are represented by a solid black line, box, and whiskers, respectively.

d) Genome-wide ratio of transitions (Ti) over transversion (Tv) with observed median displayed. Data is categorized on aNEC / aNET status. The median, interquartile range (IQR), and 1.5x the IQR are represented by a solid black line, box, and whiskers, respectively.

e) Frequency of Tandem Duplications, Insertions, Inversions, Deletions and interchromosomal translocations are indicated per sample with observed median per variable displayed. Data is categorized on aNEC / aNET status. The median, interquartile range (IQR), and 1.5x the IQR are represented by a solid black line, box, and whiskers, respectively.

f) Total number of observed structural variants (TAF ≥ 0.1), categorized on aNEC / aNET status. The median, interquartile range (IQR), and 1.5x the IQR are represented by a solid black line, box, and whiskers, respectively.

g) Mutational consequences of genomic variants (SNV, MNV and InDel) overlapping genes using Ensembl Variant Effect Predictor (VEP). Data is categorized on aNEC / aNET status.

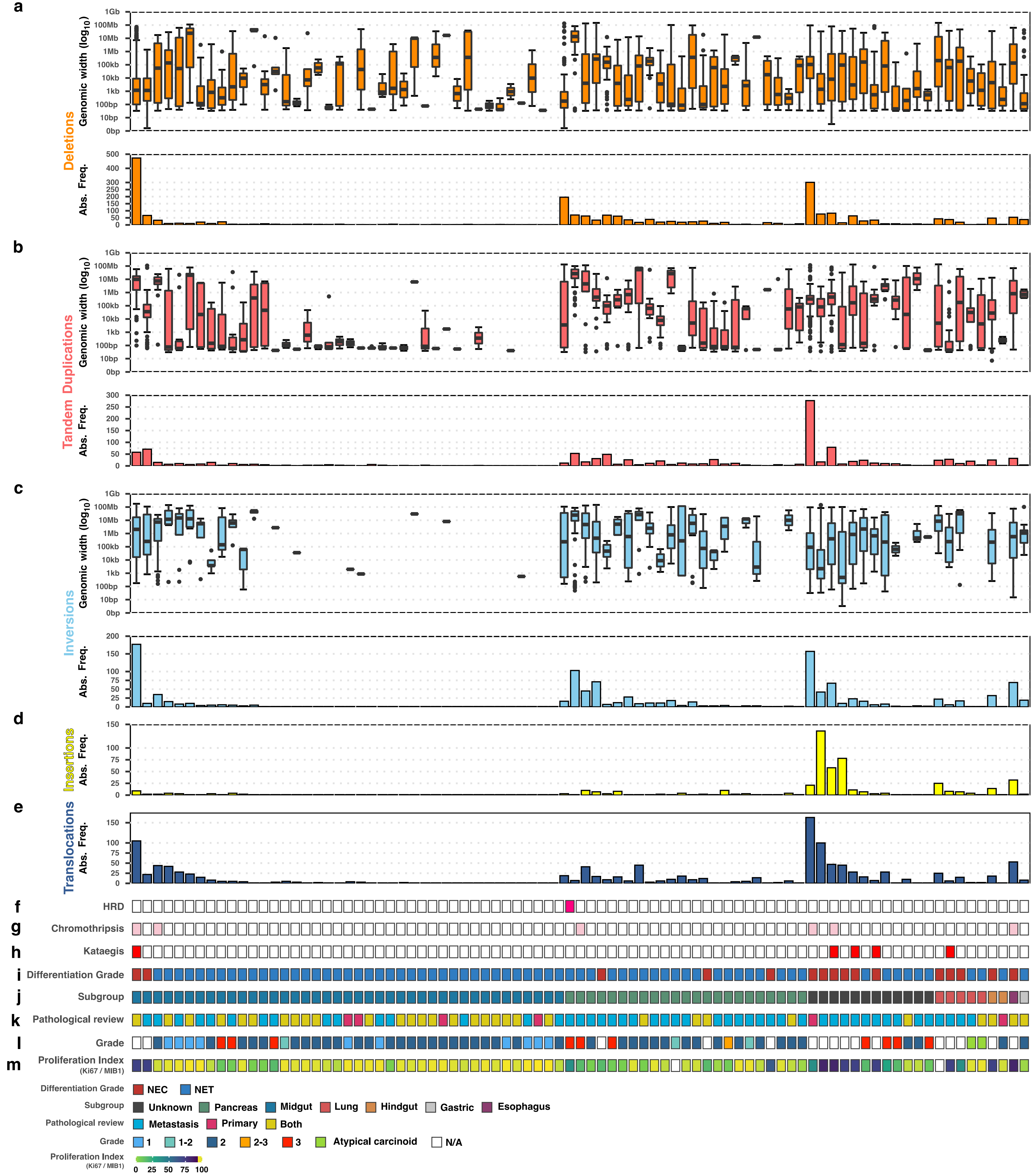

Suppl. Figure 3 - Overview of the distribution of somatically-acquired structural variants.
Overview of the genomic sizes and numbers of structural variants present in the aNEN cohort (*n* = 85). Samples are sorted based on primary localization and decreasing number of total observed structural variants over all categories (deletions, tandem duplications, inversions, translocations and insertions).

a) Track displays boxplots representing the genomic width of deletions; Y-axis is in $\log_{10}$-scale. Lower track displays the total number of observed deletions. The median, interquartile range (IQR), and 1.5x the IQR are represented by a solid black line, box, and whiskers, respectively.

b) Track displays boxplots representing the genomic width of tandem duplications; Y-axis is in $\log_{10}$-scale. Lower track displays the total number of observed tandem duplications. The median, interquartile range (IQR), and 1.5x the IQR are represented by a solid black line, box, and whiskers, respectively.

c) Track displays boxplots representing the genomic width of inversions; Y-axis is in $\log_{10}$-scale. Lower track displays the total number of observed inversions. The median, interquartile range (IQR), and 1.5x the IQR are represented by a solid black line, box, and whiskers, respectively.

d) Total number of observed translocations.

e) Total number of observed insertions.

f) Status of homologous recombination deficiency (HRD), as determined by CHORD; aNEN with *BRCA1/2*-associated HRD are shown in darkpink.

g) Presence of chromothripsis; aNEN with chromothripsis are shown in pink.

h) Presence of kataegis; aNEN with ≥1 kataegis events are shown in red.

i) Differentiation grade of the aNEN; mNEC in red, aNET in blue.

j) Primary localization of the aNEN.

k) Origin of the respective pathological record used to determine differentiation grade and proliferation index.

l) Differentiation grade based on the pathological record: 1 (sky-blue), 1-2 (teal), 2 (dark blue), 2-3 (orange), 3 (red), atypical carcinoid (green) and not available (N/A; white).

m) Proliferation index (KI67 / MIB1) from 0 to 100 based on the pathological record.

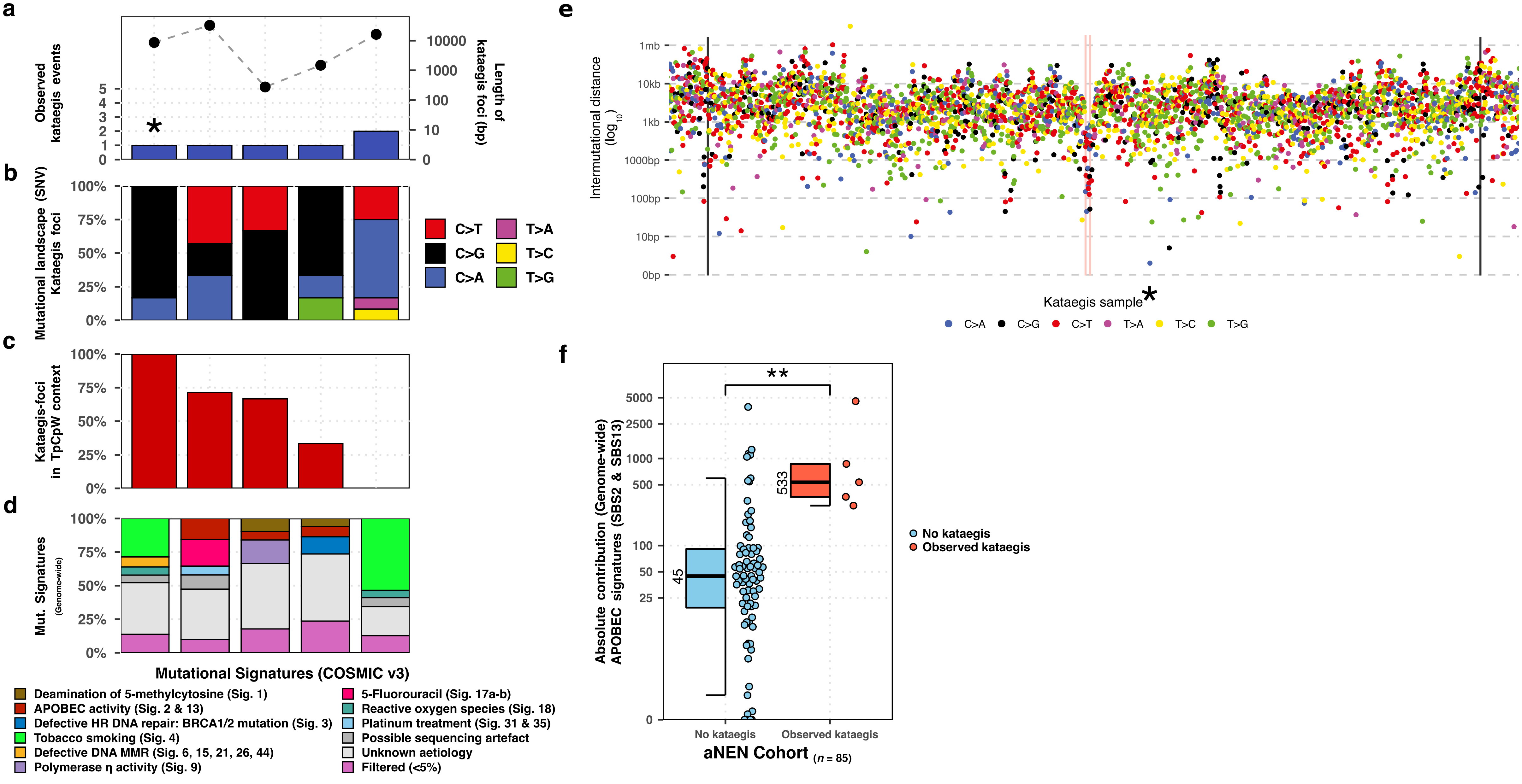

Suppl. Figure 4 - Observed kataegis events within the aNEN cohort.

a) Number of observed kataegis foci in the aNEN cohort (found in 5 distinct samples, blue bars) and the respective cumulative genomic width of all observed kataegis foci per sample (right y-axis; black points).

b) Relative frequency of SNV categories found in all observed kataegis foci per sample.

c) Relative frequency of SNV in observed kataegis foci with APOBEC-related TpCpW mutational context. W stands for T or A bases.

d) Genome-wide relative contribution to mutational signatures (COSMIC v3) in kataegis events for the respective aNEN sample.

e) Representation of a single kataegis foci on chromosome 8 within a single respective sample (highlighted with * in **a**). SNV (colored on pyrimidine mutations) are shown with relative genomic distances (in $\log_{10}$) to neighboring SNV. Observed kataegis focus on chromosome 8 is highlighted with a transparent red background.

f) Absolute mutational contribution of APOBEC COSMIC (v3) signatures (2 & 13) in base pairs for samples without (n = 80) and with observed kataegis foci (n = 5). Statistical significance was tested with a two-sided Wilcoxon rank-sum test (p = 0.0015) and is denoted with * ≤ 0.05, ** ≤ 0.01 and *** ≤ 0.001. The median, interquartile range (IQR), and 1.5x the IQR are represented by a solid black line, box, and whiskers, respectively.

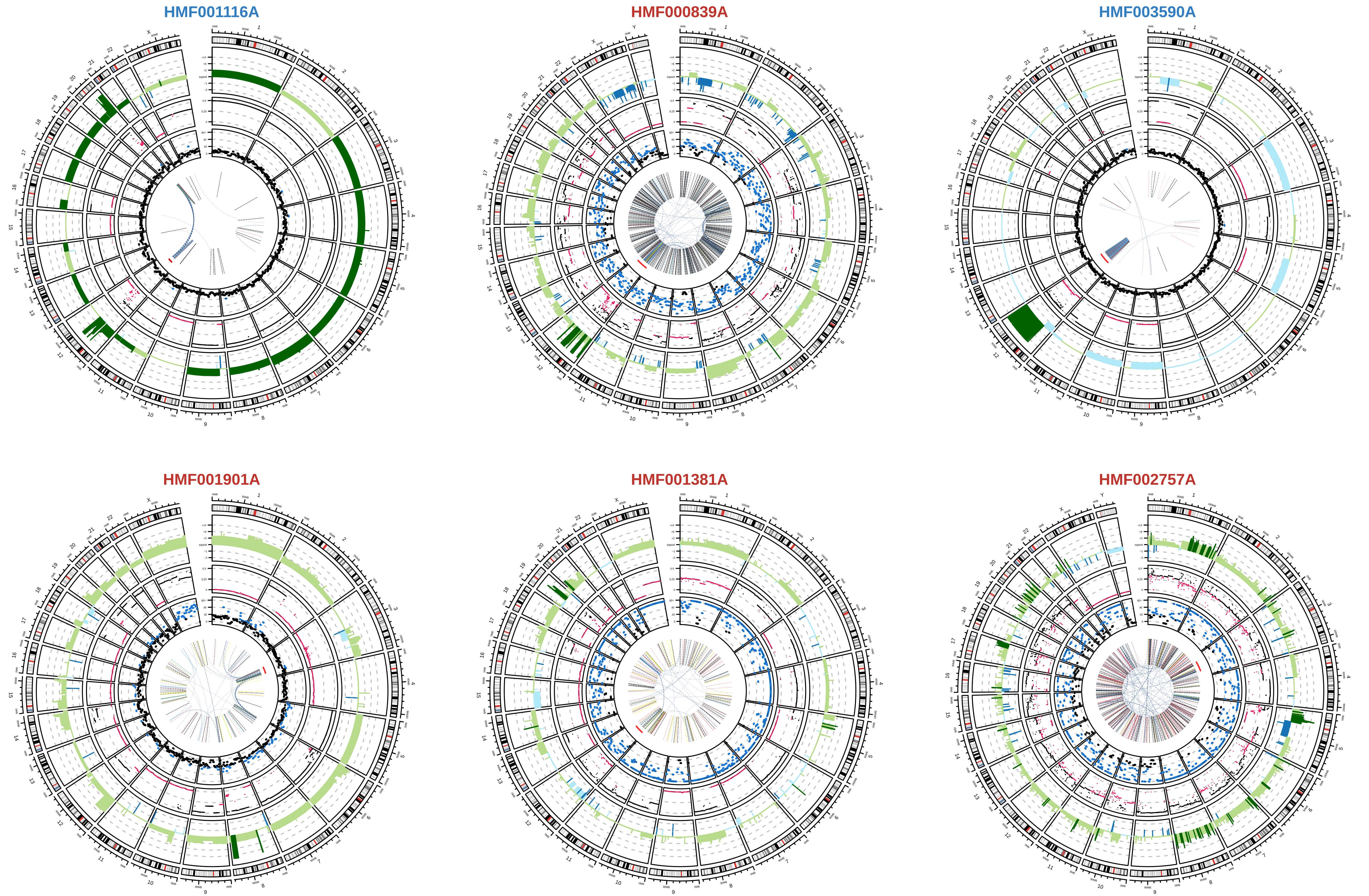

Suppl. Figure 5 - Genomic overview of aNEN displaying chromothripsis-like events.

Genomic representations of the chromothripsis-harboring aNEN (*n* = 6). The outer track displays the genomic ideogram, the second-outer track displays copy number profiles (amplification in light green; deep amplification beyond sample-specific threshold (GISTIC2) in dark green, deletions in blue; deep deletions beyond sample-specific threshold (GISTIC2) in dark blue). The third track displays TC%-corrected lower allele-frequency (LAF) values of individual copy number segments (LAF ≤ 0.33 in pink; LAF ≥ 0.33 in black). The fourth track displays the number of mutations per 5 Mbp, ranging from 0 to 60+; bins with ≥ 20 mutations are highlighted in blue. The fifth track highlights the regions harboring chromothripsis in a red line. The innermost track displays the breakpoints of the structural variants; interchromosomal translocations in dark blue, deletions in gray, insertions in yellow, inversion in light blue and tandem duplications in red. Samples are colored per NEC (in red) and NET (in blue) status.

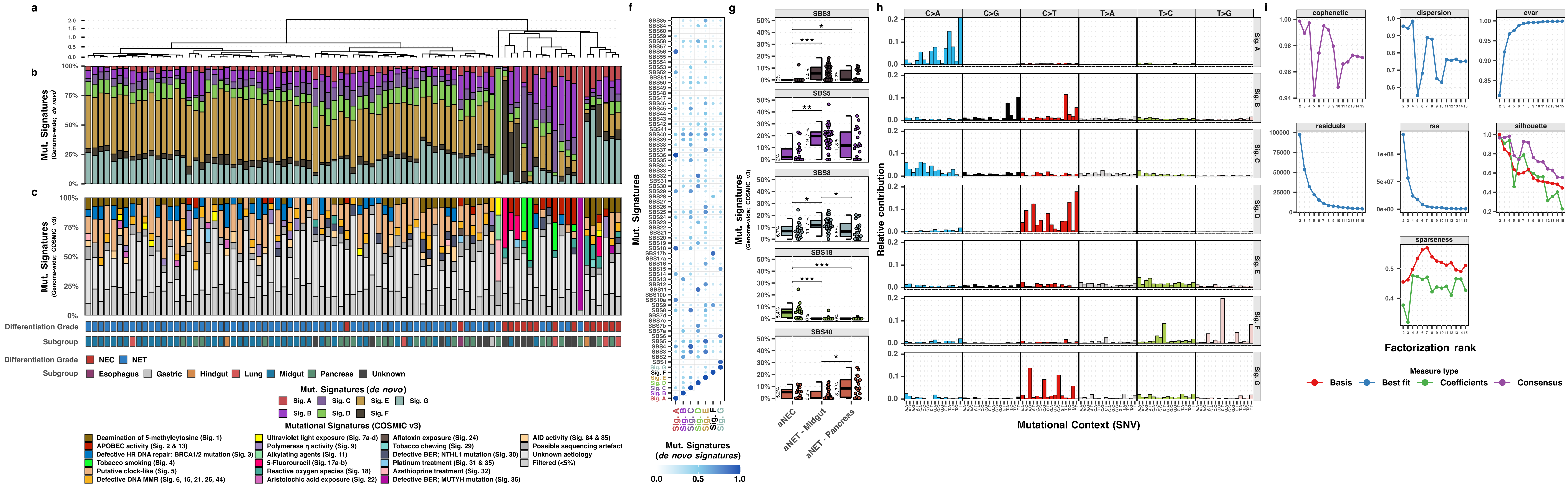

Suppl. Figure 6 - *de novo* mutational signatures assessment on aNEN.

Assessment and comparison of extracted *de novo* single base substitution mutational signatures (*n* = 7; Sig. A - G) using non-negative matrix factorization (NMF) within the aNEN cohort (*n* = 85) against the known COSMIC (v3; *n* = 67) signatures.

a) Distance of the aNEN samples based on the unsupervised clustering (Ward.D; Euclidean distance; distances plotted in $\log_{10}$-scale) of the relative contribution of the seven *de novo* mutational signatures. aNEN are subsequently sorted based on this clustering.

b) Relative genome-wide contribution of the extracted de novo single base substitution mutational signatures (*n* = 7; Sig. A - G; upper track), per aNEN.

c) Relative genome-wide contribution of extract COSMIC SBS signatures (v3; *n* = 67; lower track), per aNEN.

d) Differentiation grade of the aNEN; aNEC in red, aNET in blue.

e) Primary localization of the aNEN; Unknown (black), Pancreas (green), Midgut (blue), Lung (red), Hindgut (orange), Gastric (grey) and Esophagus (purple).

f) Cosine similarity of the de novo mutational signatures against the known COSMIC v3 signatures (*n* = 67).

g) Overview of the statistically-significant SBS signatures (COSMIC v3; two-sided Wilcoxon rank-sum test with BH-correction; *p*-adjusted (*q*) ≤ 0.05) between each of the three major subgroups: aNEC (*n* = 16), midgut-derived aNET (*n* = 39) and pancreas-derived aNET (*n* = 20). The median, interquartile range (IQR), and 1.5x the IQR are represented by a solid black line, box, and whiskers, respectively. Statistical significance is denoted by *** (*q* ≤ 0.001), ** (*q* ≤ 0.01), * (*q* ≤ 0.05) and . (*q* ≤ 0.1). The statistically significant differences are SBS3 (aNEC vs. aNET - Midgut: *q* = 0.000338, aNEC vs. aNET - Pancreas: *q* = 0.036), SBS5 (aNEC vs. aNET - Midgut: *q* = 0.00134), SBS8 (aNEC vs. aNET - Midgut: *q* = 0.021, aNET - Midgut vs. aNET - Pancreas: *q* = 0.472), SBS18 (aNEC vs. aNET - Midgut: *q* = 0.00000567, aNEC vs. aNET - Pancreas: *q* = 0.000140), SBS40 (aNET - Midgut vs. aNET - Pancreas: *q* = 0.0126)

h) Trinucleotide mutational contexts of the seven extracted *de novo* signatures.

i) NMF quality metrics using between two to fifteen ranks over 1000 iterations.

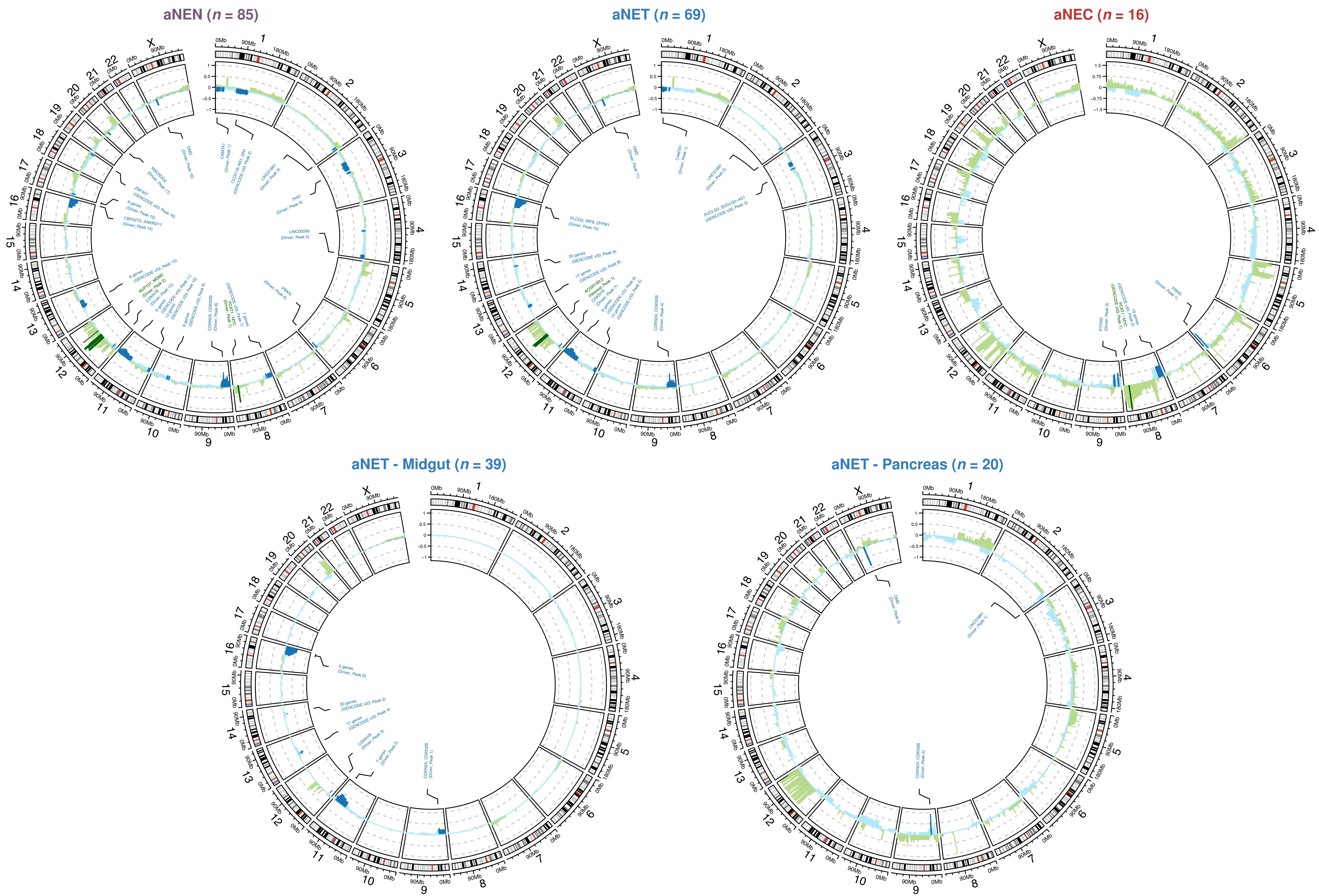

Suppl. Figure 7 - Copy-number overview of aNEN cohort and subpopulations with re-occurring and focal amplifications and deletion highlighted (GISTIC2) and unbiased driver gene analysis. Circosplots with ideogram of recurrent copy-number aberrations as detected by GISTIC2 per sub-population (as shown above each circosplot). G-scores are depicted on the y-axis. Regions with amplifications (G-score > 0) are depicted in green and deletions (G-score < 0) in blue. Regions with significant (and recurring) copy-number aberrations ($q \leq 0.1$) are denoted with a darker shade of green or blue, respective of amplification or deletion. Per region, the foci of maximal amplification or deletion (focal peaks; $q \leq 0.1$) are denoted in the inner track; the peak identifiers with associated genes are also denoted and presented in **Suppl. Data 1**.

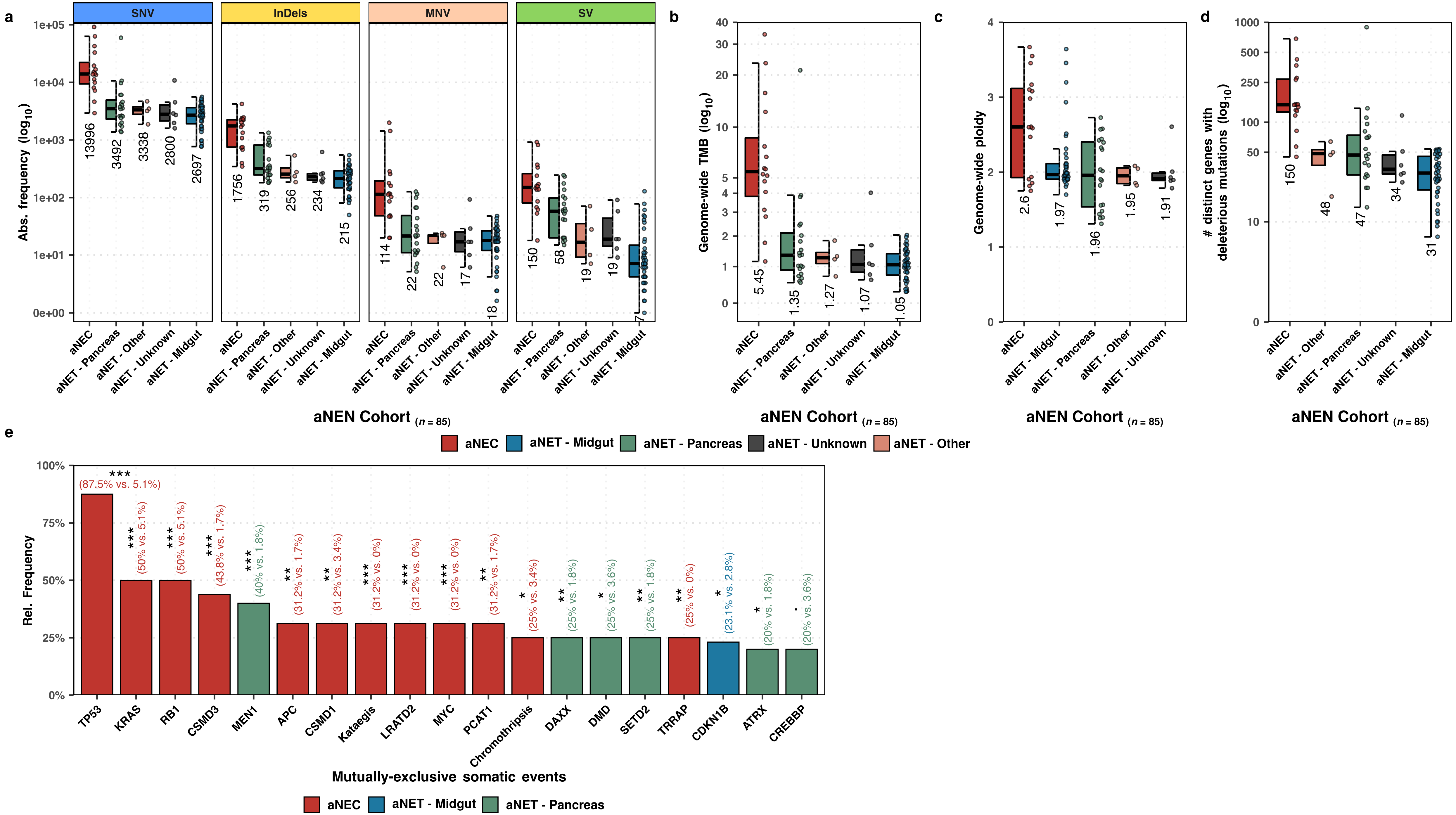

Suppl. Figure 8 - Genomic characteristics per differentiation grade (NEC/NET) and primary localization within aNET.

a) Number of SNV, InDels, MNV and SV per whole-genome aNEN with observed median per variable displayed. Data is categorized on aNEC (n = 16) and distinct aNET (n = 69) subgroups based on primary localization. The median, interquartile range (IQR), and 1.5x the IQR are represented by a solid black line, box, and whiskers, respectively.

b) Tumor mutational burdens (genome-wide; $\log_{10}$), with observed median per variable displayed. Data is categorized on aNEC (n = 16) and distinct aNET (n = 69) subgroups based on primary localization. The median, interquartile range (IQR), and 1.5x the IQR are represented by a solid black line, box, and whiskers, respectively.

c) Mean genome-wide ploidy, with observed median per variable displayed. Data is categorized on aNEC (n = 16) and distinct aNET (n = 69) subgroups based on primary localization. The median, interquartile range (IQR), and 1.5x the IQR are represented by a solid black line, box, and whiskers, respectively.

d) Number of genes harboring somatic coding mutations, with observed median per variable displayed. Data is categorized on aNEC (n = 16) and distinct aNET (n = 69) subgroups based on primary localization. The median, interquartile range (IQR), and 1.5x the IQR are represented by a solid black line, box, and whiskers, respectively.

e) Mutational enrichment of mutant genes (mutations and copy-number alterations) and large-scale events (kataegis and chromothripsis) of one major group over the two other major groups; aNEC (n = 16), pancreas- and midgut-derived aNET (n = 59). Statistical significance was tested using a one-sided Fisher's Exact Test with BH correction; significance is denoted by *** (q ≤ 0.001), ** (q ≤ 0.01), * (q ≤ 0.05) and . (q ≤ 0.1). In addition, the relative frequency of samples within the tested subgroup (as shown by the color of the bar) harboring a coding mutation and/or deep copy-number alteration vs. the other two subgroups is presented.

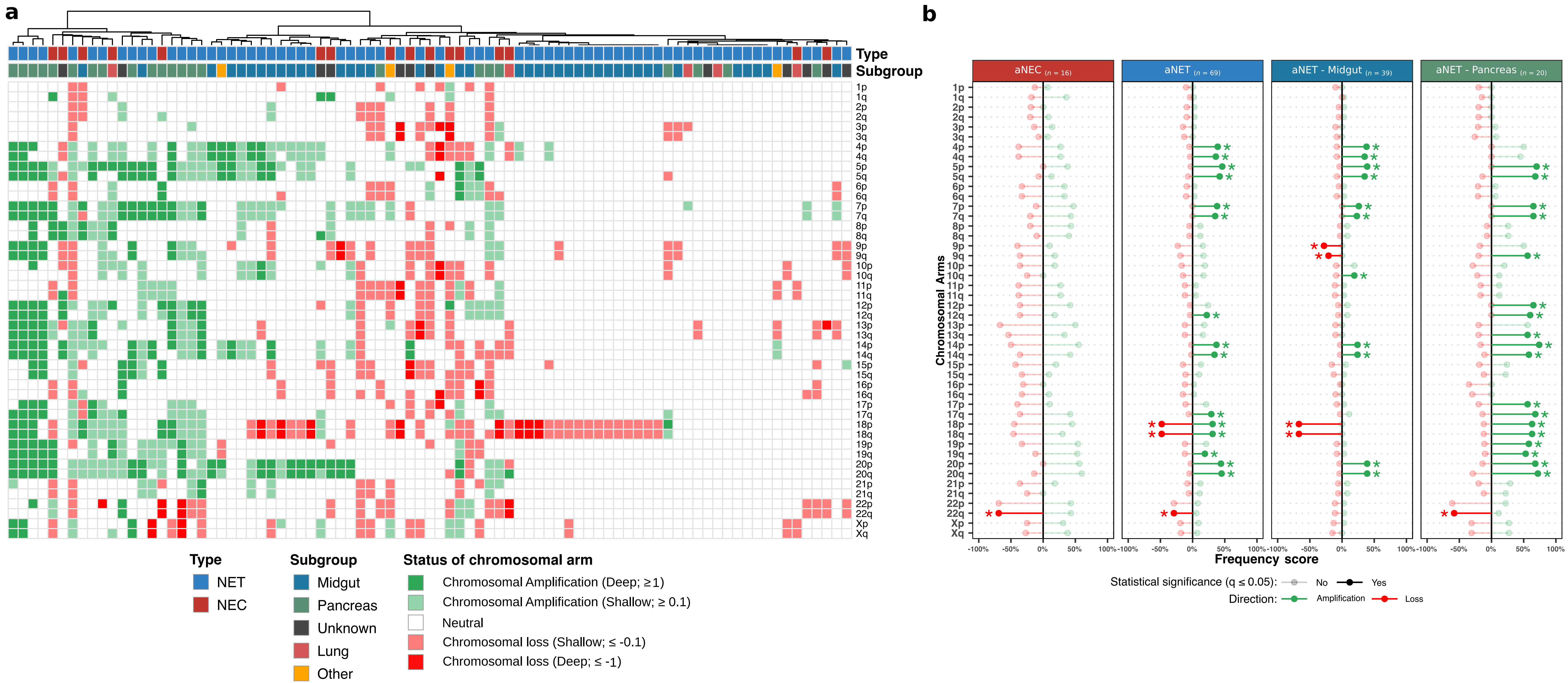

Suppl. Figure 9 - Copy-number aberrations of chromosomal arms per differentiation grade (NEC/NET) and primary localization within aNET.

a) Unsupervised clustering (Euclidean distances, Ward.D2 method) of the aNEN samples (n = 85) based on the categorization of chromosomal arm copy-number aberrations (based on GISTIC2 value per arm). Top color-bars depict the differentiation grade of the aNEN (aNEC in red, aNET in blue) and their primary localization.

b) Overview of the relative frequency of samples with amplifications (green) and losses (red) per arm within the given subgroup. Statistically significant ($q \leq 0.05$) arm-level copy-number aberrations are depicted with an asterisk whilst the non-significant events are shown as transparent.
