# Supplementary material for: The genomic landscape of 85 advanced neuroendocrine neoplasms reveals subtype-heterogeneity and potential therapeutic targets
Source: Nat Commun. 2021 Jul 29;12:4612. doi: 10.1038/s41467-021-24812-3 (PMC8322054; doi:10.1038/s41467-021-24812-3)
Supplement: Supplementary file 3 — Descriptions of Additional Supplementary Files [file 41467_2021_24812_MOESM3_ESM.pdf]

Descriptions of Additional Supplementary Files

Supplementary data 1
Description: Overview of all data presented and quantified in this manuscript.
