# Supplementary material for: The genomic landscape of 85 advanced neuroendocrine neoplasms reveals subtype-heterogeneity and potential therapeutic targets
Source: Nat Commun. 2021 Jul 29;12:4612. doi: 10.1038/s41467-021-24812-3 (PMC8322054; doi:10.1038/s41467-021-24812-3)
Supplement: Supplementary file 5 — Reporting Summary [file 41467_2021_24812_MOESM5_ESM.pdf]

nature research

Corresponding author(s): Dr. Bianca Mostert

Last updated by author(s): Apr 30, 2021

Reporting Summary

Nature Research wishes to improve the reproducibility of the work that we publish. This form provides structure for consistency and transparency in reporting. For further information on Nature Research policies, see our Editorial Policies and the Editorial Policy Checklist.

Statistics

For all statistical analyses, confirm that the following items are present in the figure legend, table legend, main text, or Methods section.

n/a	Confirmed	
☐	☒	The exact sample size (*n*) for each experimental group/condition, given as a discrete number and unit of measurement
☐	☒	A statement on whether measurements were taken from distinct samples or whether the same sample was measured repeatedly
☐	☒	The statistical test(s) used AND whether they are one- or two-sided<br>*Only common tests should be described solely by name; describe more complex techniques in the Methods section.*
☐	☒	A description of all covariates tested
☐	☒	A description of any assumptions or corrections, such as tests of normality and adjustment for multiple comparisons
☐	☒	A full description of the statistical parameters including central tendency (e.g. means) or other basic estimates (e.g. regression coefficient) AND variation (e.g. standard deviation) or associated estimates of uncertainty (e.g. confidence intervals)
☐	☒	For null hypothesis testing, the test statistic (e.g. *F*, *t*, *r*) with confidence intervals, effect sizes, degrees of freedom and *P* value noted<br>*Give P values as exact values whenever suitable.*
☒	☐	For Bayesian analysis, information on the choice of priors and Markov chain Monte Carlo settings
☒	☐	For hierarchical and complex designs, identification of the appropriate level for tests and full reporting of outcomes
☐	☒	Estimates of effect sizes (e.g. Cohen's *d*, Pearson's *r*), indicating how they were calculated

Our web collection on statistics for biologists contains articles on many of the points above.

Software and code

Policy information about availability of computer code

Data collection

Code availability:
Next to the initial processing workflows and software which are available at https://github.com/hartwigmedical/, any additional custom code and scripts used within this study (processing, analysis and visualization) have been deposited on Bitbucket under the GPL-3.0 License: https://bitbucket.org/ccbc/dr-036_mnen/

The data collection and processing is also detailed within the M&M of the manuscript (with references):

Alignment, somatic mutation detection and in silico tumor cell percentage estimation were performed in a uniform manner as detailed by Priestley et al. (2019). Briefly, paired-end sequencing reads were aligned against the human reference genome (GRCh37) using BWA-mem (v0.7.5a). Duplicate reads were marked and small insertion and deletions (InDels) were realigned using GATK IndelRealigner (v3.4.46). Prior to somatic SNV and InDel variant calling, base qualities were recalibrated using GATK BQSR (v3.4.46). Somatic SNV, InDels and MNV were called by Strelka (v1.0.14) using the matched peripheral blood WGS sample for matched-normal variant calling.

Additional in-depth settings and optimizations of the HMF pipeline are described by Priestley et al. (2019) and tools are available at https://github.com/hartwigmedical/.

The somatic mutations (SNV, InDels and MNV) were further annotated with Ensembl Variant Effect Predictor (VEP, version 99, cache 99_GRCh37) using GENCODE (v33) annotations in tandem with the dbNSFP plugin (version 3.5, hg19) for gnomAD population frequencies. SIFT and PolyPhen-2 scoring was applied for additional functional effect prediction.

During downstream analysis, we only retained SNV, InDels and MNV which passed all of the following heuristic filters; default Strelka filters

(PASS-only), gnomAD exome (ALL) allele frequency < 0.001, gnomAD genome (ALL) < 0.005, not present in ≥ 5 samples from the Hartwig Medical Foundation germline panel-of-normals (GATK Haplotyper) and not present in ≥ 3 samples from the Hartwig Medical Foundation Strelka-specific somatic blacklist.

Putative protein-altering (coding) or high-impact (e.g. splicing) mutations were aggregated per sample and gene by selecting the most deleterious annotated effect (from VEP) on any known overlapping gene-wise transcript (except those transcripts flagged as retained intron and nonsense mediated decay). In addition, structural variants with a Tumor Allele Frequency (TAF) ≥ 0.1 that overlapped only partly with the respective coding sequences (i.e. not all exons of the respective gene), were annotated as 'Structural Variant' mutations. Multiple coding mutations and/or SV per gene were annotated as 'multiple mutations'.

Discovery of somatic structural variants (SV), copy-number alterations and in-frame fusions of EWSR1 was performed using the GRIDDS (v2.9.3), PURPLE (v2.47) and LINX suite (v2.47). During the downstream analyses, we only retained somatic structural variants passing all default QC filters (PASS-only) and with an upstream and/or downstream TAF ≥ 0.1.

Data analysis

Patient cohort and study procedures
Patients with mNEN were recruited under the study protocol (NCT01855477) of the Center for Personalized Cancer Treatment (CPCT) within the CPCT-02 and the DRUP (NCT02925234) studies. This CPCT-02 protocol was approved by the medical ethical committee (METC) of the University Medical Center Utrecht. Patients were eligible for inclusion if the following criteria were met: 1) age ≥ 18 years; 2) locally advanced or metastatic solid tumor; 3) indication for new line of systemic treatment with registered anti-cancer agents; 4) safe biopsy according to the intervening physician. All patients provided written informed consent before any study procedure. The study procedures consisted of the collection of matched peripheral blood samples for reference DNA and image-guided percutaneous biopsy of the metastatic lesion. For the current study, patients were included for biopsy between May 10th 2016 and July 17th 2018 resulting in a cohort of 85 distinct patients from 13 Dutch hospitals (Supplementary table 1).

Collection of the pathological records and generalization of pre-treatment(s).
Primary tumor characteristics of the 85 included mNEN patients were checked within the nationwide network and registry of histo- and cytopathology in the Netherlands (PALGA).75
From PALGA, we collected the differentiation grade and proliferation index (Ki67 / MIB1) based on the pathological records of the patient-specific primary and/or any metastatic lesion. If more than one pathological report was available, we chose to include the report most close in date, but always prior to, the biopsy for the CPCT 02 study.

The pre-treatment(s) of mNEN patients prior to the collection and sequencing of the metastatic biopsy has been collected and generalized on treatment classification. Out of all included mNEN patients (n = 85), 26 patients received pre-treatment according to our clinical records.
Collection, sequencing and processing of mNEN biopsies.
Blood samples were collected in CellSave preservative tubes (Menarini-Silicon Biosystems, Huntington Valley, PA, USA) and shipped by room temperature to the central sequencing facility at the Hartwig Medical Foundation. Tumor samples were fresh-frozen in liquid nitrogen directly after the procedure and send to a central pathology tissue facility. Tumor cellularity was estimated by assessing a hematoxylin-eosin (HE) stained 6 micron section. Subsequently, 25 sections of 20 micron were collected for DNA isolation. DNA was isolated with an automated workflow (QiaSymphony) using the DSP DNA Midi kit for blood and QIAsymphony DSP DNA Mini kit for tumor samples according to the manufacturer's protocol (Qiagen). DNA concentration was measured by Qubit™ fluorometric quantitation (Invitrogen, Life Technologies, Carlsbad, CA, USA). DNA libraries for Illumina sequencing were generated from 50-100 ng of genomic DNA using standard protocols (Illumina, San Diego, CA, USA) and subsequently whole-genome sequenced in a HiSeq X Ten system using the paired-end sequencing protocol (2x150bp) for both the metastatic tumor and matched blood sample.

Subsequent alignment, somatic mutation detection and in silico tumor cell percentage estimation were performed in a uniform manner as detailed by Priestley et al. (2019). Briefly, paired-end sequencing reads were aligned against the human reference genome (GRCh37) using BWA-mem (v0.7.5a). Duplicate reads were marked and small insertion and deletions (InDels) were realigned using GATK IndelRealigner (v3.4.46). Prior to somatic SNV and InDel variant calling, base qualities were recalibrated using GATK BQSR (v3.4.46). Somatic SNV, InDels and MNV were called by Strelka (v1.0.14) using the matched peripheral blood WGS sample for matched-normal variant calling.

Additional in-depth settings and optimizations of the HMF pipeline are described by Priestley et al. (2019) and tools are available at https://github.com/hartwigmedical/.

The somatic mutations (SNV, InDels and MNV) were further annotated with Ensembl Variant Effect Predictor (VEP, version 99, cache 99_GRCh37) using GENCODE (v33) annotations in tandem with the dbNSFP plugin (version 3.5, hg19) for gnomAD population frequencies. SIFT and PolyPhen-2 scoring was applied for additional functional effect prediction.

During downstream analysis, we only retained SNV, InDels and MNV which passed all of the following heuristic filters; default Strelka filters (PASS-only), gnomAD exome (ALL) allele frequency < 0.001, gnomAD genome (ALL) < 0.005, not present in ≥ 5 samples from the Hartwig Medical Foundation germline panel-of-normals (GATK Haplotyper) and not present in ≥ 3 samples from the Hartwig Medical Foundation Strelka-specific somatic blacklist.

Putative protein-altering (coding) or high-impact (e.g., splicing) mutations were aggregated per sample and gene by selecting the most deleterious annotated effect (from VEP) on any known overlapping gene-wise transcript (except those transcripts flagged as retained intron and nonsense mediated decay). In addition, structural variants with a Tumor Allele Frequency (TAF) ≥ 0.1, as calculated by PURPLE and GRIDSS84, that overlapped only partly with the respective coding sequences (i.e., not all exons of the respective gene), were annotated as 'Structural Variant' mutations. Multiple coding mutations and/or SV per gene were annotated as 'multiple mutations'.

Discovery of somatic structural variants (SV), copy-number alterations and in-frame fusions of EWSR1 was performed using the GRIDDS, PURPLE and LINX suite.84 During the downstream analyses, we only retained somatic structural variants passing all default QC filters (PASS-only) and with an upstream and/or downstream TAF ≥ 0.1.

Mean read coverages of the reference and tumor samples were calculated using Picard Tools (v1.141; CollectWgsMetrics) based on GRCh37. Genomic and coding tumor mutational burden (TMB) was calculated as previously described by van Dessel, van Riet et al. (2019).

Discovery of genes under evolutionary selection

We performed a dN/dS analysis on somatic mutations (SNV and InDels) using dndscv (v0.0.1.0) on respective genome sequences and transcript annotations using a custom transcript database based on ENSEMBL88 Genes (v99)/GENCODE (v33) annotations. We performed a dN/dS analysis over the entire NEN cohort (n = 85) and four separate dN/dS analysis on the major subgroups (mNEC; n = 16, NET; n = 69, mNET-midgut; n = 39 and mNET-pancreas; n = 20). Genes-of-interest were selected based on the statistical significance, corrected for multiple hypothesis testing (Benjamini-Hochberg), which integrated all mutation types (missense, nonsense, essential splice-site mutations and InDels; qglobal_cv ≤ 0.1) and/or without InDels (qallsubs_cv ≤ 0.1).

Detection and annotation of recurrent copy-number alterations

To detect recurrent copy-number alterations, we performed a GISTIC289 (v2.0.23) analysis over the entire mNEN cohort and, again, four separate GISTIC2 analysis on the major subgroups (mNEC, mNET and pancreas- and midgut-derived mNET).

The GISTIC2 was performed using the following settings:

Genes were annotated to GISTIC2 peaks (q ≤ 0.1) based on the following strategy;

GISTIC2 focal peaks (all_lesions.conf_95.txt) were overlapped to genes (from verified and manually annotated loci, no pseudogenes or read-throughs and from standard chromosomes; n = 36574) from GENCODE (GRCh37; v33), taking into consideration only the genes overlapping with at least 100 base pairs within the detected GISTIC2 peak.

If a GISTIC2 focal peak overlapped with multiple GENCODE genes, a combined database containing known drivers detected in a metastatic pan-cancer dataset (CPCT-02), COSMIC Cancer Gene Census (v85), OncoKB Cancer Gene Census (June 2019), Martincorena et al. (2017)87 and Priestley et al. (2019) were used to further pinpoint the possible target gene(s) (n = 1272), e.g. if a GISTIC2 peak overlapped both PTEN and near-adjacent non-driver gene, only PTEN would be chosen as possible gene. The list of all overlapping GENCODE (v33) genes per GISTIC2 peak can be found in supplementary table 1.

If no overlapping genes were found, GISTIC2 peaks were annotated with the nearest GENCODE (v33) protein-coding gene (n = 19988).

Mutational signature analysis

Mutational signatures based on the trinucleotide contexts of SNVs was performed, using the MutationalPatterns package (1.10.0) and as previously described. The 96 Single Base Substitution (SBS) mutational signatures (COSMIC v3) as established by Alexandrov et al. (2019), (matrix Sij; i = 96; number of trinucleotide motifs; j = number of signatures) were downloaded from COSMIC (as deposited on May 2019). The proposed etiology of each SBS signature was derived from Alexandrov et al. (2019), Petljak et al. (2019), Angus et al. (2019) and Christensen et al. (2019).

In addition, de novo mutational signature analysis by MutationalPatterns (1.10.0) was performed based on the max. number of relevant signatures as assessed using the NMF R package (v0.21.0) with 1000 iterations (supplementary figure 6d). By comparing the cophenetic correlation coefficient, residual sum of squares and silhouette, we opted to generate seven custom de novo signatures. Custom signatures were correlated to existing (COSMIC v3) mutational signatures using cosine similarity.

Detection of chromothripsis

Shatterseek (v0.4) using default parameters was used to detect chromothripsis-like events. As input, we used the rounded absolute copy numbers (as derived by PURPLE) and structural variants with an TAF ≥ 0.1 at either end of the breakpoint. The male sex chromosome (chrY) was excluded. The criteria for a chromothripsis-like event were based on the following criteria: a) total number of intra-chromosomal structural variants involved in the event ≥25; b) max. number of oscillating CN segments (2 states) ≥7 or max. number of oscillating CN segments (3 states) ≥14; c) total size of chromothripsis event ≥20 megabase pairs (Mbp); d) satisfying the test of equal distribution of SV types ($p > 0.05$); and e) satisfying the test of non-random SV distribution within the cluster region or chromosome ($p \leq 0.05$).

Classification of homologous recombination deficiency genotypes

To determine Homologous Recombination Deficiency (HRD) due to possible loss-of-function of BRCA1 and/or BRCA2 (amongst others), we utilized the Classifier for Homologous Recombination Deficiency with default settings (CHORD; v2.0). CHORD uses a random-forest approach to classify samples into HR-deficient / HR-proficient categories. Briefly, we make use of CHORD31; a random-forest based classifier designed to classify samples with evidence of HRD (BRCA1-type, BRCA2-type or otherwise) by using all the information captured within all the somatic small mutations and somatic structural variants of whole-genome sequenced samples. If a sample contains sufficient HRD-related genomic scars (structural variants) and additional markers for HRD, that sample will be classified as HR-deficient (HRD).

Detecting enrichment of mutant genes within major subgroups.

To determine the enrichment of mutant genes within our major subgroups (mNEC, midgut- and pancreas-derived mNET), we generated a list of potential driver genes based on captured genes through our dN/dS (q ≤ 0.1) analysis and/or present within the focal amplification and deletion peaks captured by GISTIC2. We extended this list by selecting genes which contained a coding mutation in ≥20% of a respective subgroup or which harbored a deep amplification or deletion in ≥20% of the respective subgroup (i.e., 20% of the respective subgroup contained coding mutations and/or ≥20% contained a copy-number alteration, irrespective of coding mutation). Using this list of genes (n = 20), we performed a one-sided (enrichment) Fisher's Exact Test with Benjamini-Hochberg correction between each pairwise comparison per major subgroup against the remaining major subgroups (e.g., mNEC vs. the combined group of midgut- and pancreas-derived mNET).

Inventory of clinically-actionable somatic alterations and putative therapeutic targets

Current clinical relevance of somatic alterations in relation to putative treatment options or resistance mechanisms and trial eligibility was determined based upon the following databases; CiViC (Nov. 2018), OncoKB (Nov. 2018), CGI (Nov. 2018) and the iClusion (Dutch) clinical trial database (Sept. 2019) from iClusion (Rotterdam, the Netherlands). The databases were aggregated and harmonized using the HMF knowledgebase-importer (v1.7). This list was manually corrected for discrepancies and subsequently, we curated the linked putative treatments for current on- and off-label mNEN and mNEN-subtype treatment options, as defined within the Netherlands by the Dutch Medicines Evaluation Board ("College ter Beoordeling van Geneesmiddelen; CBG).

For manuscripts utilizing custom algorithms or software that are central to the research but not yet described in published literature, software must be made available to editors and reviewers. We strongly encourage code deposition in a community repository (e.g. GitHub). See the Nature Research guidelines for submitting code & software for further information.

Data

Policy information about availability of data

All manuscripts must include a data availability statement. This statement should provide the following information, where applicable:

- Accession codes, unique identifiers, or web links for publicly available datasets
- A list of figures that have associated raw data
- A description of any restrictions on data availability

The WGS and corresponding clinical data used in this study was made available by the Hartwig Medical Foundation (Dutch nonprofit biobank organization) after signing a license agreement stating data cannot be made publicly available via third party organizations. Therefore, the data are available under restricted access and can be requested upon by contacting the Hartwig Medical Foundation (https://www.hartwigmedicalfoundation.nl/applying-for-data/) under the accession code DR-036. Within this manuscript, we furthermore made use of the actionable gene-variant and associated drug databases of CiViC (01-Nov-2018; https://civicdb.org/downloads/01-Nov-2018/01-Nov-2018-ClinicalEvidenceSummaries.tsv), OncoKB (Nov. 2018; https://www.oncokb.org/actionableGenes), CGI (Nov. 2018; https://www.cancergenomeinterpreter.org/biomarkers) and the iClusion (Dutch) clinical trial database (Dec. 2020) from iClusion (Rotterdam, the Netherlands; Suppl. Table 1). The remaining data are available within the Article, Supplementary Information or available from the authors upon request.

Field-specific reporting

Please select the one below that is the best fit for your research. If you are not sure, read the appropriate sections before making your selection.

[x] Life sciences   [ ] Behavioural & social sciences   [ ] Ecological, evolutionary & environmental sciences

For a reference copy of the document with all sections, see nature.com/documents/nr-reporting-summary-flat.pdf

Life sciences study design

All studies must disclose on these points even when the disclosure is negative.

Sample size	No a priori sample size test was performed. We performed analysis on all the distinct mNEN samples which had been successfully sequenced within the CPCT-02 consortium (n = 85). Considering the rare occurrence of this malignancy, we considered this to be representative of mNEN as a whole. This is the largest (WGS) repository to date with previous (m)NEN cohort-analysis being performed on as small as ~5-25 samples.
Data exclusions	If multiple WGS samples were available from the same patient(s), these were excluded and only the first biopsy was used in order to prevent inflation of observed measurements. In addition, if the clinical records of the patients (among other biopsy location, prior therapy, sample identifiers etc.) were not all available, we did not take along these samples. This is further detailed within the manuscript and main figure 1.<br><br>In addition, following review of the associated pathological reports we've excluded a single sample prior to analysis.
Replication	A duplicate (WGS) cohort of mNEN is not available for replication/validation. We compared previous findings within the field of (m)NEN genomics and found them to be concordant with our cohort in to testify for the quality of our cohort as no other surrogate was available.
Randomization	N/A, this study makes no use of separate experimental groups. Inclusion criteria of patients within the CPCT-02 and DRUP studies are detailed within the manuscript.
Blinding	Blinding was not relevant as investigators specifically wished to make use of sample meta-data (biopsy location, prior therapy) within the various analysis to, for example, compare pancreas vs. midgut-derived mNEN. All results are based upon WGS-derived metrics as detailed within the manuscript.

Reporting for specific materials, systems and methods

We require information from authors about some types of materials, experimental systems and methods used in many studies. Here, indicate whether each material, system or method listed is relevant to your study. If you are not sure if a list item applies to your research, read the appropriate section before selecting a response.

Materials & experimental systems

n/a	Involved in the study	
[x]	[ ]	Antibodies
[x]	[ ]	Eukaryotic cell lines
[x]	[ ]	Palaeontology and archaeology
[x]	[ ]	Animals and other organisms
[ ]	[x]	Human research participants
[ ]	[x]	Clinical data
[x]	[ ]	Dual use research of concern

Methods

n/a	Involved in the study	
[x]	[ ]	ChIP-seq
[x]	[ ]	Flow cytometry
[x]	[ ]	MRI-based neuroimaging

Human research participants

Policy information about studies involving human research participants

Population characteristics	The mNEN cohort is represented by 37 females and 48 males with a median age of 62 (Q(uartile)1-Q3: 57-68) and 61 (Q1-Q3: 56-68) years, at time of biopsy respectively (Figure 1c). In total, 69 NET and 16 NEC were included.<br><br>Patients were eligible for inclusion if the following criteria were met: 1) age ≥ 18 years; 2) locally advanced or metastatic solid tumor; 3) indication for new line of systemic treatment with registered anti-cancer agents; 4) safe biopsy according to the intervening physician.<br><br>The primary tumor location in the midgut was most common (n = 41, 48%), followed by pancreas (n = 23, 27%) and unknown (n = 12, 14%). Most of the tumor biopsies were taken from liver metastases, and a minority from relapses at the primary site.
Recruitment	Patients with mNEN were recruited under the study protocol (CPCT-02 Biopsy Protocol, ClinicalTrial.gov no. NCT01855477) of the Center for Personalized Cancer Treatment (CPCT) within the CPCT-02 and the DRUP (The Drug Rediscovery Protocol (DRUP Trial), ClinicalTrial.gov no. NCT02925234) studies. All analyzed biopsies were taken prior to treatment within the DRUP trial.
Ethics oversight	The CPCT-02 (NCT01855477) and DRUP (NCT02925234) clinical studies, which were approved by the medical ethical committees (METC) of the University Medical Center Utrecht and the Netherlands Cancer Institute, respectively

Note that full information on the approval of the study protocol must also be provided in the manuscript.

Clinical data

Policy information about clinical studies

All manuscripts should comply with the ICMJEguidelines for publication of clinical research and a completedCONSORT checklist must be included with all submissions.

Clinical trial registration	Patients with mNEN were recruited under the study protocol (CPCT-02 Biopsy Protocol, ClinicalTrial.gov no. NCT01855477) of the Center for Personalized Cancer Treatment (CPCT) within the CPCT-02 and the DRUP (The Drug Rediscovery Protocol (DRUP Trial), ClinicalTrial.gov no. NCT02925234) studies.
Study protocol	We have added the full study protocols of the CPCT-02 trial as this was the major source of all samples (83 out of 85 samples). The full protocol for the DRUP-study has not been published publicly, but is available upon (reasonable) request.
Data collection	Patients were accrued at multiple hospitals throughout the Netherland. All patients included in this manuscript were accrued between May 10th 2016 and July 17th 2018.
Outcomes	This study performed additional in-depth analysis on the mNEN patients captured within the CPCT-02 and DRUP studies.
